# Relationship between Neutrophil-to-Lymphocyte Ratio and Inflammatory Markers in Sickle Cell Anaemia Patients with Proteinuria

**DOI:** 10.3390/medsci4030011

**Published:** 2016-07-29

**Authors:** Mathias Abiodun Emokpae, Austin Aruomaren, Evarista Osime

**Affiliations:** Department of Medical Laboratory Science, School of Basic Medical Sciences, College of Medical Sciences, University of Benin, Benin, 300001, Nigeria; Austin.aruomaren@uniben.edu (A.U.); evaristaosime@yahoo.com (E.O.)

**Keywords:** lymphocyte, neutrophil, proteinuria, sickle cell anaemia

## Abstract

The renal functions and structure in sickle cell anaemia (SCA) patients may be affected by chronic haemodynamic changes and consequences of vaso-occlusive events in the renal medulla. Few reports on neutrophil-to-lymphocyte (NLR) and platelet-to-lymphocyte (PLR) ratios in SCA patients in Africans exist in the literature. This study correlates the values of NLR and PLR with measured traditional inflammatory markers in SCA patients with and without proteinuria and impaired kidney function (defined in this study as estimated glomerular filtration rate (eGFR), less than 60 mL/min/1.73 m^2^. Full blood count, C-reactive protein (CRP), and fibrinogen were assayed in 150 SCA patients and 50 control subjects using Coulter Haematology analyser (CELL DYE 37000) and ELISA method, respectively. The NLR and PLR were calculated by dividing absolute neutrophil or platelet counts by absolute lymphocyte count. Fibrinogen, CRP, NLR, and PLR increased progressively (*p <* 0.001) in SCA patients with or without proteinuria, with the highest values seen in those with impaired renal function. NLR correlated positively with CRP and fibrinogen in SCA patients without proteinuria (*p <* 0.001), with proteinuria (*p <* 0.001), and impaired renal function (*p <* 0.05). A positive relationship was also observed between NLR and fibrinogen in the control subjects. The need to determine cut-off values for these leukocyte ratios to be used in identifying those patients at risk and in the general management of SCA patients is suggested.

## 1. Introduction

The renal functions and structure in sickle cell anaemia (SCA) patients may be affected by chronic haemodynamic changes and consequences of vaso-occlusive events in the renal medulla [1]. Inflammatory events are common in SCA; neutrophil-to-lymphocyte (NLR) and platelet-to-lymphocyte (PLR) ratios—as indicators of sub-clinical inflammation—have rarely been investigated in SCA patients with proteinuria in Nigeria. We recently reported on the association of NLR, PLR, and their association with the atherogenic index of plasma in sickle cell nephropathy (SCN) [2].

Sickle cell disease (SCD) is an inherited autosomal recessive genetic disease of haemoglobin (Hb) caused by a mutation of the β-globin gene of haemoglobin, which leads to the substitution of a single nucleotide from thymine to adenine in haemoglobin, resulting in the amino acid valine instead of glutamic acid [3]. This change is responsible for the alteration in the properties of the haemoglobin tetramer, with a tendency to polymerize in the deoxygenated state [4]. The disorder is characterized by the polymerization of protein, changing the normal flexible biconcave shaped red blood cells (RBCs) into stiff, rigid, sickle cells. Polymerization within the red cell causes it to deform, become rigid, obstruct blood flow, and produce acute and chronic tissue damage because of poor perfusion [5]. Polymerization in sickle cell disease has pleiotropic effects, which include vaso-occlusion, strokes, haemolytic anaemia, increased infection, and ischaemic organ damage. Researchers have directly related the rate of polymerization to the severity of the vaso-occlusive episodes observed in SCA patients [6].

The vascular endothelium plays a critical role in vaso-occlusion and ischaemic organ damage by several mechanisms. Evidence indicates that vascular endothelium is damaged in SCD, thereby triggering a systemic inflammatory process [5]. Tissue factors are expressed in activated endothelium and play key roles in the recruitment of leukocytes and the promotion of thrombosis at sites of vascular inflammation [7].

Acute painful episodes are the most frequent complication of SCD. However, SCN has also been reported as a complication of SCD and is indicated by the presence of sickled erythrocytes in the renal medulla that result in decreased medullary blood flow, ischaemia, and papillary necrosis in the kidneys. These pathologic changes could result in tubular and glomerular functional disturbances that affect blood pressure regulation, water and electrolyte metabolism [8]. Any injury or endothelial cell death in the renal medulla impairs the endothelial cells from performing their critical traditional homeostatic functions. Chronic haemolytic events in SCD often result in the accumulation of Hb and heme, leading to Hb/heme toxicity [9,10].

A systemic inflammatory process is thought to arise in sickle cell patients during the polymerization of the red cells, which interact with the vascular endothelium and impede blood flow. Inflammatory cells, neutrophils, and megakaryocytes are recruited, causing neutrophilia and possible thrombocytosis, leading to complications in SCD [11]. Serum level of C-reactive protein (CRP)—the most commonly-assessed marker of acute and chronic inflammation—was associated with increased risk of vaso-occlusive crisis in SCA patients, thereby resulting in acute chest syndrome [12]. Other reports have associated a strong link between high sensitivity CRP and vaso-occlusion in paediatric SCA patients [13]. The intravital microscopy studies on SCD mice revealed the presence of some adhesive molecules such as vascular cell adhesion module (VCAM 1, intercellular adhesion module (ICAM) 1, and P-selectin that aided the interaction between sickle red blood cells, leukocytes, and endothelial cells [9].

In recent years, several indicators derived from the peripheral blood, such as the NLR and PLR, have been investigated as useful prognostic markers for sub-clinical inflammation in several diseases such as cardiovascular, renal, and gastrointestinal diseases and cancers [2,14,15]. It was reported that the leukocyte ratios may predict patients’ response to inflammatory injury, with neutrophils increasing as a result of stress and a decrease in the lymphocyte population induced by apoptosis [16]. The ratios, therefore, could be used to identify those subjects who do not have the physiological reserve to withstand the inflammatory events [16]. Since the leukocyte ratios have been associated with inflammation in several other disease conditions, we have decided to correlate these leukocyte ratios with proteinuria in SCD subjects.

There is, however, paucity of report on NLR in SCA patients with proteinuria in the African population. This study seeks to correlate values of NLR and PLR with the measured traditional inflammatory markers in SCA patients with and without proteinuria and impaired kidney function (defined in this study as estimated glomerular filtration rate (eGFR) less than 60 mL/min/1.73 m^2^).

## 2. Patients and Methods

This study was conducted in Kano, Nigeria. All subjects involved in this study also gave informed consent. One hundred and fifty subjects participated in this study. These 150 subjects were divided into four groups. Group A (*n =* 50) comprised individuals with normal haemoglobin (Hb AA), and they were routinely selected from the general population (control). Group B (*n =* 68) were SCA subjects without macroalbuminuria. Group C (*n =* 17) were SCA subjects with macroalbuminuria, and Group D (*n =* 15) were subjects with impaired renal function.

### 2.1. Ethical Consideration

The protocol for this study was reviewed and approved by the ethical committee of the Aminu Kano Teaching Hospital, Kano (AKTH/EC/2007/017), and all participants gave informed consent before samples were collected.

### 2.2. Sample Collection and Preparation

Eight mL of venous blood were collected aseptically, and 2 mL dispensed into potassium ethylene diamine tetraacetic acid (K_2_EDTA) at a concentration of 2.0 mg/mL of blood. Three mL was dispensed into another tube containing 3.8% sodium citrate for fibrinogen assay. The remaining 3 mL was emptied into a plain container and allowed to clot at room temperature. This was centrifuged at 3000 rpm for 10 min using a Compact II centrifuge (Pittsburgh USA) and the serum was separated into another plain container and was kept frozen until analysed. The sample collected in K_2_EDTA was used for the complete blood count (haematocrit, Hb levels, total leukocyte count, platelet count, absolute neutrophil, lymphocyte, and platelet counts).

### 2.3. Analytical Methods

Complete blood count was determined using the Coulter Counter Automated Haematology Analyser (CELL DYE 3700; Abbott diagnostics, Wiesbaden, Germany). NLR was calculated by dividing the value of absolute neutrophil count by absolute lymphocyte count, while PLR was calculated by dividing absolute platelet count by absolute lymphocyte count. The serum CRP and plasma fibrinogen were assayed using reagent kits supplied by Sigma (MO, USA) and Anogen (ON, Canada), respectively. Glomerular filtration rate (GFR) was calculated using the Cockcroft–Gault formula [17]. Urinalysis was initially done by dipstick technique using early morning urine specimens, while 24 h urinary protein was determined in those subjects who were positive for macro-albuminuria using sulphosalysilic acid colorimetric technique.

### 2.4. Statistical Analysis

The results obtained were presented as mean ± standard error of the mean (SEM) and were analysed using a statistical software package (SPSS version 16, IBM, IL, USA). One-way analysis of variance (ANOVA) was used to compare test results (between SCA patients with or without proteinuria and impaired renal function) and with the controls. Correlation analysis was also carried out using GraphPad Prism 6 (Cal, USA to test the relationship between NLR and PLR with measured inflammatory markers.

## 3. Results

The 150 subjects that participated in this study were between the ages of 20 and 36 years. The mean age of the control group was 26.86 ± 3.0 years, the mean age in group B (SCA patients without macroalbuminuria) was 21.86 ± 3.0 years, the mean age in group C (SCA patients with macroalbuminuria) was 20.9 ± 4.0 years, and in group D (SCA patients with impaired kidney function) it was 32.7 ± 3.2 years (Table 1).

The mean levels of haematocrit (PCV), Hb, and total leukocyte count decreased progressively in SCA patients with and without macroalbuminuria, as well as in those with impaired renal function compared with control subjects. Platelet and absolute neutrophil count levels increased in SCA patients with or without macroalbuminuria and in those with impaired renal function compared with control subjects. In this study, inflammatory markers were compared between the groups, and an ANOVA analysis of the results revealed that NLR and PLR increased in SCA patients without proteinuria, with proteinuria, and patients with impaired kidney function when compared with control subjects, as shown in Table 2. The CRP level significantly increased in SCA patients with macroalbuminuria (*p <* 0.05) and in those with impaired kidney function when compared with control subjects. However, no significant difference was observed when compared with SCA patients without macroalbuminuria. Additionally, fibrinogen level was significantly increased (*p <* 0.05) in SCA patients with impaired kidney function when compared with control subjects and the difference was insignificant when compared with other groups (Table 2).

Table 3 shows the correlation between NLR and other traditional inflammatory markers and urine protein concentrations; the results revealed a significant positive correlation (*p <* 0.001) between NLR and CRP in SCA patients with or without proteinuria, and in SCA patients with impaired renal function (*p <* 0.05). Similarly, NLR correlated positively (*p <* 0.001) with fibrinogen in controls and in SCA patients with or without proteinuria. A significant correlation (*p <* 0.05) was also observed between NLR and fibrinogen in SCA patients with impaired renal function. In addition, NLR correlated positively with urinary protein levels in SCA patients with macroalbuminuria (*p <* 0.05) and SCA patients with impaired renal function (*p <* 0.02, Table 3).

## 4. Discussion

The values of NLR and PLR increased progressively in SCA patients with or without macroalbuminuria, with the highest values seen in SCA patients with impaired renal function. The NLR correlated positively with CRP and fibrinogen levels, which are traditional inflammatory markers in the studied groups. The observed result is consistent with recent studies, which evaluated the leukocyte ratios in patients with various disease conditions other than SCA [18]. Increased NLR was significantly associated with insulin resistance in type 2 diabetes mellitus and was considered a reliable predictive marker of insulin resistance [18]. An association between raised NLR and increased concentrations of pro-inflammatory cytokines was reported [19]. The association between the leukocyte ratios and outcomes in various critically ill patients has also been reported [20]. It was suggested that the ratios may be predictive of patients’ response to inflammatory insult, with neutrophils increasing due to stress and lymphocytes decreasing in population due to apoptosis [21]. Lymphocytes are involved in the regulation of an appropriate inflammatory response and decrease in population either due to apoptosis, down-regulation, or cellular DNA damage, which could exacerbate adverse inflammatory conditions [22]. It was suggested that an elevated leukocyte ratio may be used to identify patients who do not have physiological reserve to withstand the inflammatory insult and low survival outcomes [20]. We recently reported a statistically significant association between these leukocyte ratios and atherogenic index of plasma lipid in SCN [2].

Kato and colleagues [23] reported that damaged endothelium and tissue exposure to pro-inflammatory cytokines and growth factors play important roles in sickle cell pathophysiology. The results from our study showed that there were increases in inflammatory markers such as NLR, PLR, CRP, and fibrinogen in SCA patients. The findings suggest that CRP and fibrinogen levels in SCA patients with or without macroalbuminuria were significantly increased when compared to controls. This result is in agreement with our previous report [24]. Our observation was partially supported by that of Aneke et al. [14], where it was reported that NLR was significantly higher in SCA patients, but not in SCA patients with macroalbuminuria and without proteinuria. The role of reperfusion injury and inflammation in the pathogenesis of SCA was reported in SCA patients [25,26]. SCA is a disease characterized by chronic haemolytic events, resulting in increased release of Hb and heme into the circulation. In normal physiological state, the potential toxic effect of these molecules is eliminated by scavenging systems involving haptoglobin (Hp) and haemopexin (Hx) that bind Hb and heme, respectively. The Hp–Hb and Hx–heme complexes are taken up by macrophages until they are cleared by their respective receptors without toxic effect. In SCD, however, the levels of Hb and heme exceed the scavenging capacity of Hp and Hx, thereby causing Hb- and heme-mediated oxidative tissue damage [9,10,27]. It has also been reported that heme acts as a pro-inflammatory molecule that can activate the Toll-like receptor 4 (TLR4), which in turn is capable of inducing the activation of endothelial cells and generating inflammatory cytokines [28].

The findings indicate that an association exists between NLR, PLR, and CRP in SCA patients with or without macroalbuminuria. However, no association between NLR, PLR, and CRP was observed in SCA patients without macroalbuminuria. Similarly, NLR was positively associated with urinary proteins in SCA patients with macroalbuminuria and impaired renal function. This suggests that in SCA patients, these simple and easily assayed parameters could be used as an adjunct to other investigations to assess the levels of inflammation in the management of patients. Further, macroalbuminuria has been regarded as sufficient evidence for SCN, since this mostly represents early stages of kidney function deterioration arising from proteinuria [29]. Sickling of the red blood cells—especially in the renal medulla—causes acute and chronic ischaemia, resulting in progressive tissue damage [29]. Several stress conditions could trigger endothelial injury or death, and these include oxidative stress, metabolic stress, endoplasmic reticulum stress, and genotoxic stress, as well as innate and adaptive immune system [9,30]. Most of these conditions have been reported in SCA patients [1,24]. Due to chronic haemolysis, SCA patients are constantly exposed to the increased generation of reactive oxygen species (ROS) catalysed by Fenton reaction, and the vessel walls are the primarily exposed tissue [9,31]. Studies have reported that oxidative stress and inflammation may contribute directly to pathophysiological events in SCA [32]. The significant association between NLR and SCA patients with impaired renal function observed in this study was at variance with that reported by Aneke et al. [14]. However, Kocyigit et al. [33] suggested that NLR might predict the progression rate of stage four chronic kidney disease (CKD) to dialysis, and that it is an easily accessible and useful marker for monitoring CKD patients in clinical practice.

In this study, the blood counts (haematocrit, Hb concentration, total leukocyte count, and absolute lymphocyte count) were significantly higher in control subjects when compared to SCA patients with or without macroalbuminuria and those with impaired renal function. It is important to note that the decreases in these blood count parameters occurred in SCA patients with or without macroalbuminuria and those with impaired renal function (Table 1). However, absolute platelet count was significantly lower in control subjects when compared with SCA patients. It is worth noting, however, that when compared among SCA patients, those with impaired renal function had a significantly higher platelet count compared to others (*p <* 0.05).

## 5. Conclusions

The NLR and PLR correlated with the measured traditional inflammatory markers in SCA patients, with values increasing in SCA patients with macroalbuminuria and the highest levels were seen in those with impaired renal function. These ratios may be superior prognostic markers to individual leukocyte indices in evaluating inflammatory insult in SCD patients if their cut-off values are known. It is suggested that these simple and easily-assayed parameters could be used as an adjunct to other investigations in the assessment of inflammation in the management of SCA patients.

## Figures and Tables

**Table 1:** Comparison of some haematological indices in sickle cell disease (SCD) patients with macroalbuminuria and chronic kidney disease.

Parameters	Hb AA Controls (Group A) *n =* 50	SCA Patients without Macroalbuminuria (Group B) *n =* 86	SCA Patients with Macroalbuminuria (Group C) *n =* 17	SCA Patients with Impaired Renal Function (Group D) *n =* 15
Age	26.86 ± 3.0 ^b^	21.86 ± 3.0 ^a^	20.9 ± 4.0 ^a^	32.7 ± 3.2 ^b^
Haematocrit (%)	38.30 ± 4.9 ^b^	20.3 ± 4.9 ^a^	19.8 ± 2.9 ^a^	18.8 ± 1.2 ^b^
Haemoglobin (g/dL)	12.10 ± 1.8 ^b^	7.10 ± 1.8 ^c^	6.4 ± 0.8 ^a^	6.2 ± 0.4 ^a^
Total leukocyte count (×10^9^/L)	6.40 ± 0.4 ^b^	2.47 ± 0.4 ^b^	2.12 ± 0.8 ^c^	2.06 ± 0.1 ^a^
Platelet count (×10^9^/L)	308 ± 98 ^a^	370 ± 98 ^c^	347 ± 82 ^c^	430 ± 120.1 ^b^
Absolute lymphocyte count (×10^9^/L)	3.40 ± 0.2 ^a^	4.0 ± 1.2 ^b^	3.0 ± 0.4	2.7 ± 0.2 ^b^
Absolute neutrophil count (×10^9^/L)	4.11 ± 1.3 ^a^	5.3 ± 1.2 ^c^	6.1 ± 0.8 ^b^	6.5 ± 0.4 ^b^
NLR	1.21 ± 0.07 ^a^	1.25 ± 0.06 ^a^	2.0 ± 0.02 ^c^	2.33 ± 0.03 ^b^
PLR	90.6 ± 8.8 ^a^	92.5 ± 4.2 ^a^	115.7 ± 5.8 ^c^	159.2 ± 5.9 ^b^

Analysis of variance (ANOVA) was used to compare means between the groups. All similar letters (superscript) indicate means that are not significantly different, while different letters indicate that means that are significantly different between the groups (a = *p >* 0.05; b = *p <* 0.001; c = *p <* 0.05). Hb AA: normal haemoglobin group; NLR: neutrophil-to-lymphocyte ratio; PLR: platelet-to-lymphocyte ratio; SCA: sickle cell anaemia.

**Table 2:** Comparison of some inflammatory markers in SCD patients with macroalbuminuria and chronic kidney disease.

Parameters	Hb AA Controls (Group A) *n =* 50	SCA Patients without Macroalbuminuria (Group B) *n =* 86	SCA Patients with Macroalbuminuria (Group C) *n =* 16	SCA Patients with Impaired Renal Function (Group D) *n =* 16
NLR	1.21 ± 0.07 ^a^	1.25 ± 0.06 ^a^	2.0 ± 0.02 ^c^	2.33 ± 0.03 ^b^
PLR	90.6 ± 8.8 ^a^	92.5 ± 4.2 ^a^	115.7 ± 5.8 ^c^	159.2 ± 5.9 ^b^
CRP (μg/mL)	0.81 ± 0.92 ^a^	1.12 ± 0.02 ^b^	1.21 ± 0.4 ^c^	1.81 ± 0.05 ^c^
Fibrinogen (mg/dL)	290 ± 30.5 ^a^	297 ± 15.2 ^a^	303 ± 6.8 ^a^	317 ± 4.1 ^c^
Proteinuria (g/dL)	0.00 ± 0.00	0.00 ± 0.00	0.08 ± 0.02^c^	0.17 ± 0.01 ^b^

ANOVA was used to compare the means between the groups. All similar letters (superscript) indicate means that are not significantly different while different letters indicate means that are significantly different between the groups (a = *p >* 0.05; b = *p <* 0.001; c = *p <* 0.05). CRP: C-reactive protein.

**Table 3:** Correlation between NLR and other inflammatory markers in SCA patients and controls.

Parameters	Hb AA Controls	SCA Patients without Macroalbuminuria	SCA Patients with Macroalbuminuria	SCA Patients with Impaired Renal Function
CRP (μg/mL)	*R* = −0.014, *p* = 0.92	*R* = 0.900, *p <* 0.0001	*R* = 0.978, *p <* 0.0001	*R* = 0.515, *p* = 0.04
Fibrinogen (mg/dL)	*R* = 0.988, *p <* 0.0001	*R* = 0.900, *p <* 0.0001	*R* = 0.978, *p <* 0.0001	*R* = 0.524, *p* = 0.04
Proteinuria (g/dL)	-	-	*R*= 0.483, *p <* 0.05	*R*= 0.575, *p <* 0.02

*R* represents Pearson *R* value, *p <* 0.05 is significant.
